# Helping the Architect

**Published:** 1919-06-14

**Authors:** 


### HOSPITAL CONSTRUCTION: THE NEW ERA.

### VIII. Helping the Architect.

The position of a hospital architect is a very exceptional one, without any close parallel in other professional spheres. Possibly the practice of a barrister who specialises in technical cases comes near to it; but the unusual reciprocity between the hospital architect and the men and women who make up the scientific and economic staff of the buildings with which that architect has to deal constitutes a mutuality of effort which can hardly be said to be closely rivalled in the legal or any other profession.

Like all professional men the hospital architect is essentially an adviser. Yet, if his career is to be valid and useful he must be for ever learning from people whom he advises. He is in one sense more of a collaborator than an adviser; but, as he is paid to advise it is clear that for all his dependence upon his employers he nevertheless has to keep the lead and to know with some surety what are the points, the occasions, upon which he necessarily becomes the receiver rather than the purveyor of advice. There is a sense, of course, in which every architect is more or less in this position of give and take. Whatever be the class of building upon which he is engaged, it is very seldom that he is given absolutely *carte blanche*. Almost every man who has money enough to order a building has some ideas which he wishes to see carried out in that building.

There is no doubt the case—the rare case—of the ignorant *parvenu* who wishing to be housed as the nobility are housed and to live as the aristocracy lives says to his architect "You know what I ought to have. I know that I don't care what I spend. Go forward without bothering me and produce the ideal home of the man I wish to become." But such cases are uncommon and are at variance with the ordinary laws of social equilibrium. Generally speaking, the fact is that the owner or promoter of any building knows something which his architect does not know about the uses of the building and trusts his architect to know more than himself about the way in which those requirements should be met. But the relationship of the hospital architect to his multiple client is much more subtle than this. The function which a hospital has to perform is one which is always alive with change. The much over-worked French phrase "*plus ça change . . .*" never had a truer meaning than as describing the ever-changing identity of hospital effort.

Now it is perfectly clear that, as we hinted in one of the preliminary articles of this series, it is impossible for any man to be a leader in the two realms of medicine and architecture at the same time. Both worlds are too big for single mortals to conquer. It is therefore certain that the architect of these buildings, while making sure of his supremacy as an architect, must rely for his efficiency in the medical and surgical aspect of his craft on that co-operative help from the actual workers of the healing profession which is always so readily offered.

We would not be misunderstood. It is not our meaning that the architect in designing a given hospital should rely for every detail of accommodation on detailed instructions from the staff. Far from it. He will probably find, if he is a man of experience in this class of work, that he knows more about most of the general and special points of hospital construction than do his employers. But how did he gain this superior knowledge? Simply by learning; and mostly by learning, not from other architects, but from the physicians, surgeons, matrons, sisters, nurses, and others, not excluding cooks, porters, and liftmen with whom he has if he is wise held consultation during his career. This little list of consultants is no doubt likely to raise a smile; but it is not one to be ridiculed. The experienced architect when he approaches the task of rebuilding a hospital will very soon find out who among the staff—meaning staff in the widest sense—have something or nothing to teach him. It is part of his very difficult business to judge in the interests of his building where he can pick up knowledge and where he cannot; and it is safe to say that no person, however humble, who has practical work to do in the routine of a hospital may not be able to help with a hint or a complaint that may lead to some useful improvement. Of course, it is among the great pioneers in the worlds of surgery and medicine that the greatest and most advancing traffic is to be made. No hospital architect should ever lose opportunities of reading all that is to be read or of hearing all that is to be heard about the newest discoveries and the newest ideas. He should never design his buildings merely on the lines of blind precedent. Every time he sets pencil to paper on a new scheme he should ask himself why he does this, that, and the other thing, why he continues the use of one device or another. In the world of hygiene everything changes, not details only, but principles; and it is only the watchful eye, the inquiring spirit, and the humility of the patient learner that can keep abreast of the ever-altering needs which subserve the one unaltering purpose.

It is here that the generosity of the medical and allied professions comes into play. These skilled men and women, anxious no doubt in the first instance for the success and supremacy of their own particular institution, will, we have always found, give freely of their special knowledge in the cause of the general advancement of the great work of hospital construction. It is by their help to the architect and the architect's ingenuity in clothing new needs in new methods of building that the enterprise of hospital development goes marching

☛ The previous articles appeared on Jan. 25, Feb. 8, March 1, 15 and 29, April 19, and May 17, p. 153.

**Hospital Construction: The New Era**—(*continued*).

along. And such co-operation can only win through to its end by mutual concessions as well as mutual gifts. Each party must recognise when the technical knowledge of the other must take the lead. Obstinacy is of no value; but purpose, which is a very different thing, is of the greatest value. A conflict between architect and doctors leads to no useful issue, but in the patient comparing of knowledge with knowledge, in the continual sharpening, by the conference of wits, of the fine edge of progress lies the secret of the advance which has been made and will continue to be made in the art and craft of designing homes of healing.

